# *In silico* approaches to discover the functional impact of non-synonymous single nucleotide polymorphisms in selective sweep regions of the Landrace genome

**DOI:** 10.5713/ajas.18.0122

**Published:** 2018-05-31

**Authors:** Donghyun Shin, Kyung-Hye Won, Ki-Duk Song

**Affiliations:** 1 Department of Animal Biotechnology, Chonbuk National University, Jeonju 54896, Korea; 2 The Molecular Genetics and Breeding Center, Chonbuk National University, Jeonju 54896, Korea

**Keywords:** Landrace, Next-generation Sequencing, Non-synonymous Single Nucleotide Polymorphism, Reproductive Capacity, Selective Sweep

## Abstract

**Objective:**

The aim of this study was to discover the functional impact of non-synonymous single nucleotide polymorphisms (nsSNPs) that were found in selective sweep regions of the Landrace genome

**Methods:**

Whole-genome re-sequencing data were obtained from 40 pigs, including 14 Landrace, 16 Yorkshire, and 10 wild boars, which were generated with the Illumina HiSeq 2000 platform. The nsSNPs in the selective sweep regions of the Landrace genome were identified, and the impacts of these variations on protein function were predicted to reveal their potential association with traits of the Landrace breed, such as reproductive capacity.

**Results:**

Total of 53,998 nsSNPs in the mapped regions of pigs were identified, and among them, 345 nsSNPs were found in the selective sweep regions of the Landrace genome which were reported previously. The genes featuring these nsSNPs fell into various functional categories, such as reproductive capacity or growth and development during the perinatal period. The impacts of amino acid sequence changes by nsSNPs on protein function were predicted using two in silico SNP prediction algorithms, i.e., sorting intolerant from tolerant and polymorphism phenotyping v2, to reveal their potential roles in biological processes that might be associated with the reproductive capacity of the Landrace breed.

**Conclusion:**

The findings elucidated the domestication history of the Landrace breed and illustrated how Landrace domestication led to patterns of genetic variation related to superior reproductive capacity. Our novel findings will help understand the process of Landrace domestication at the genome level and provide SNPs that are informative for breeding.

## INTRODUCTION

The recently developed high-throughput and cost-effective genotyping techniques allow the thorough exploration of genetic variation in domestic animals. In particular, whole-genome sequencing is a powerful approach for detecting massive amounts of single nucleotide polymorphisms (SNPs) in genome-wide sequence data. One of the strategies for studying genetic variation is to detect the selective sweep signatures based on patterns of linkage disequilibrium (LD) [1], which was proposed by Smith and Haigh [2], and other researchers have expanded and applied it [3–6]. Wang et al [7] performed a relative extended haplotype homozygosity (REHH) test to detect selective sweep regions of the Landrace genome using genotyping by genome sequencing. The genetic signature for selection of body size investigated by estimating the XP-EHH statistic in the Yucatan miniature pig [8]. Whole-genome re-sequencing of Jeju black pig (JBP) and Korean native pigs (which live on the Korean peninsula) were performed to identify signatures of positive selection in JBP, the true and pure Korean native pigs [9]. Studies of selective sweeps in pigs have revealed strong selection signatures associated with genes underlying economic traits such as the body length, disease resistance, pork yield, muscle development, and fertility [10,11].

Diverse types of variants, e.g. copy number variations, insertion/deletion (InDel) and structural variations, have been identified in the selective sweep regions of the Landrace genome [7]. Unlike many SNPs are phenotypically neutral, non-synonymous SNPs (nsSNPs) that are located in protein-coding regions and lead to amino acid substitutions in the corresponding protein product might have functional impacts and play a role in biological processes through altering the protein structure, stability, or function, these variations are often strongly associated with several phenotypes [12]. In the case of pigs, previous studies reported the different polymorphic patterns of nsSNPs in the Toll-like receptor genes between European wild boars and domestic pigs [13].

In this study, we aimed to identify nsSNPs in the selective sweep regions of the Landrace genome that might be related to superior reproductive capacity or growth and development during the perinatal period, and gene networks that were enriched in Landrace genome. Finally, impact of amino acid changes by nsSNPs on protein function was also investigated using *in silico* bioinformatic tools.

## MATERIALS AND METHODS

### Sample preparation and whole-genome re-sequencing

**Figure 1:**
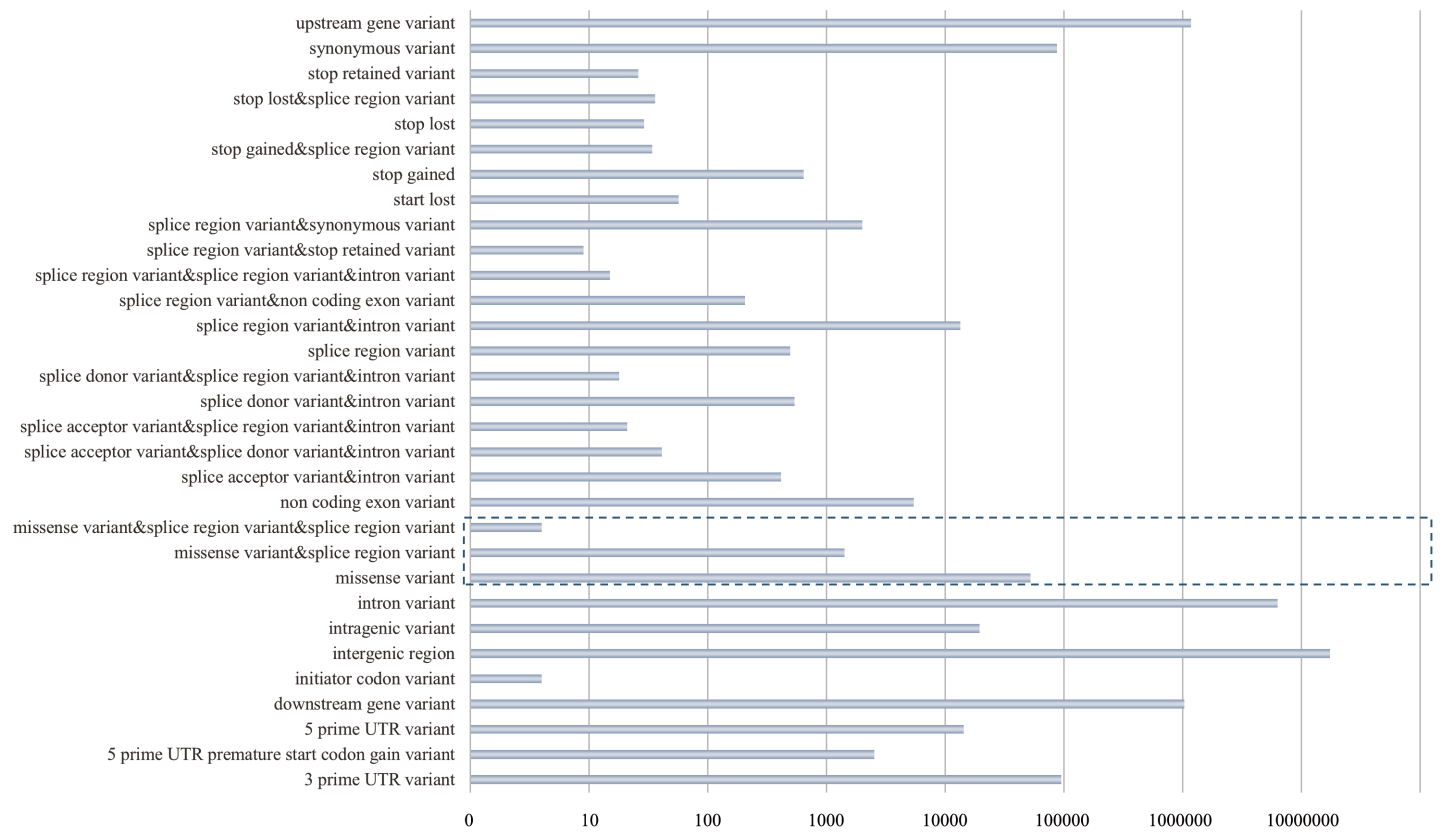
Functional classification of total single nucleotide polymorphisms (SNPs) from 40 pig whole-genome sequences (16 Yorkshire, 14 Landrace, and 10 wild boar). After SNP calling, all filtered SNPs (a total of 26,240,429 SNPs) were annotated using an SNP annotation tool, SnpEff version 4.1a (reference), and the Ensembl *Sus scrofa* gene set version 75 (Sscrofa10.2.75). Through SnpEff, we divided all SNPs into 31 functional classes containing non-synonymous SNPs (missense variants), as shown in this figure. The dotted line box in this figure indicates non-synonymous SNPs.

In this study, a whole-genome sequence data set consisting of 14 Landrace (Danish), 16 Yorkshire (Large White) pigs, and 10 wild boars, were obtained from the NCBI Sequence Read Archive database (SRP047260). FastQC software [14] were used to perform a quality check on raw sequence data. Using Trimmomatic-0.32 [15], potential adapter sequences were removed before sequence alignment. Paired-end sequence reads were mapped to the pig reference genome (*Sscrofa* 10.2.75) from the Ensembl database using Bowtie2 [16] with the default settings. For downstream processing and variant calling, following software packages were used: Picard tools (http://broadinstitute.github.io/picard/), SAMtools [17], and Genome Analysis Toolkit (GATK) [18]. “CreateSequenceDictionary” and “MarkDuplicates” Picard command-line tools were used to read reference FASTA sequences for writing bam files with only a sequence dictionary and to filter potential polymerase chain reaction duplicates, respectively. Using SAMtools, index files were created for the reference and bam files. Local realignment of sequence reads was performed to correct misalignment due to the presence of small insertions and deletions using GATK “Realigner-TargetCreator” and “IndelRealigner” arguments. In addition, base quality score recalibration was performed to obtain accurate quality scores and to correct the variation in quality with machine cycle and sequence context. For calling variants, GATK “UnifiedGenotyper” and “SelectVariants” arguments were used with the following filtering criteria. All variants with i) a Phred-scaled quality score of less than 30; ii) read depth less than 5; iii) MQ0 (total count across all samples of mapping quality zero reads) >4; or iv) a Phred-scaled p-value using Fisher’s exact test of more than 200 were filtered out to reduce false-positive calls due to strand bias. “vcf-merge” tools of VCFtools [19] were used to merge all of the variants calling format files for the 40 samples. Additionally, tri-allelic SNPs were excluded, and all filtered SNPs on autosomes (a total of 26,240,429 SNPs) were annotated using an SNP annotation tool, SnpEff version 4.1a and the Ensemble *Sus scrofa* gene set version 75 (Sscrofa10.2.75). 53,998 nsSNPs (missense variants) were identified on autosomes from 40 sets of pig whole-genome data (Figure 1). Then, certain SNPs due to poor genotyping quality were removed; 4,174 SNPs were excluded based on Hardy-Weinberg equilibrium testing (p≤ 0.000001). In addition, a total of 19,002 SNPs with a minor allele frequency of <0.05 were excluded. After genomic data quality control, there were 30,822 SNPs for downstream analysis.

### Population structure analysis

**Figure 2:**
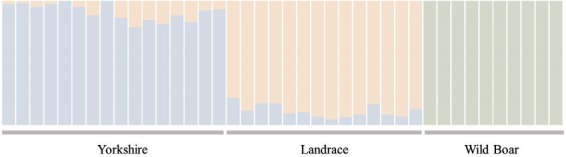
Population structure analysis using STRUCTURE. Each individual is represented by a vertical bar, and the length of each colored segment in each of the vertical bars represents the proportion contributed by ancestral populations (K = 3).

Population structure analysis was performed to infer the population structure of the 40 pigs with whole-genome sequence data. The program STRUCTURE (https://web.stanford.edu/group/pritchardlab/structure.html) was used to evaluate the extent of substructure among the 40 individuals belonging to three pig breeds. Bayesian clustering analysis implemented in STRUCTURE (version 2.3.4) was used to estimate the population structure using 30,822 nsSNPs from the whole-genome sequencing data of the 40 pigs [20]. An initial burn-in of 10,000 iterations were followed by 10,000 iterations for parameter estimation was sufficient to ensure the convergence of parameter estimates. To estimate the number of populations (the K parameter of STRUCTURE), the dataset was analyzed by allowing for the values of K = 3 (Figure 2).

### Identify nsSNPs in Landrace selective sweep regions

**Figure 3:**
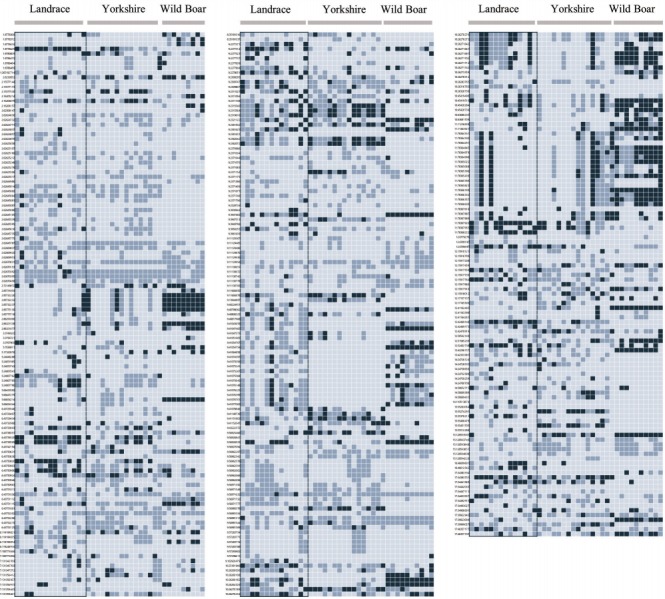
Genotypes of 345 non-synonymous single nucleotide polymorphisms (SNPs) in Landrace selective sweep regions. The genotype patterns of 345 non-synonymous SNPs in the selective sweep regions of the Landrace genome are represented by a heat map. The colors of the boxes represent the genotypes of each of the 40 individuals from the whole-genome sequencing data. Dark blue indicates that the genotypes of both the alleles were the same as that of the minor allele. Blue boxes indicate that one of the two alleles was the same as the minor allele and the other was the same as the major allele. Sky blue means that the genotypes of both alleles were the same as that of the major allele. The left side of the figure shows a list of each SNP name, which consists of the chromosome, position, and minor allele type. The gray box at the bottom of the figure indicates the three breeds.

A previous study identified 269 selective sweep regions of the Landrace genome using the REHH test (p-value≤0.01), which was used to detect the recent positive selection signatures by evaluating how LD decays across the genome 7. A total of 261 of 269 selective sweep regions of the Landrace genome were on autosomes, and 345 nsSNPs belonged to 55 Landrace selective sweep regions were identified (Figure 3). Overall, 345 nsSNPs in 55 selective sweep regions of the Landrace genome belonged to 90 genes, and gene function 64 of total 90 genes were discovered. Gene ontology (GO) network analysis was performed using ClueGO [21] to infer the biological meaning of the genes related to nsSNPs in Landrace selective sweep regions.

### Predicting damaging amino acid substitutions of non-synonymous SNPs specific to the Landrace breed

In this study, the functional effects of nsSNPs were predicted using the following in silico algorithms: sorting intolerant from tolerant (SIFT) [22] and polymorphism phenotyping v2 (Polyphen-2) [23]. Total 345 nsSNPs in 55 selective sweep regions of the Landrace genome were analyzed using SIFT. NsSNPs with less than 0.05 of SIFT score, which was regarded as deleterious, were used for PolyPhen-2 ver. 2.2.2 (http://genetics.bwh.harvard.edu/pph2/) analysis to predict the influence of an amino acid change on the structure and function of a protein by using specific empirical rules [23]. From the results of Polyphen-2 analysis, nsSNPs were classified into probably damaging, possibly damaging, and benign based on their scores (ranging from 0 to 1); if Polyphen-2 score for nsSNPs was more than 0.95, nsSNPs were considered to be “probably damaging”, while for values between 0.5 and 0.95, they were regarded as “possibly damaging”. The scores below 0.5 were classified as “benign”. In this study, probably damaging and possibly damaging SNPs were judged as to have strong effects on protein function.

If the SIFT score of each SNP was less than 0.05, the SNP was regarded as being deleterious, which could strongly affect protein function. Additionally, we performed PolyPhen-2 (version 2.2.2) analysis to predict the influence of an amino acid change on the structure and function of a protein by using specific empirical rules [23]. Amino acid sequences corresponding to nsSNPs of interest from the Ensembl database were obtained to perform PolyPhen-2 analysis.

## RESULTS

### DNA sequencing, data preprocessing, and genetic variant calling

A total of 26,240,429 SNPs were extracted on autosomes from the whole-genome sequences of the 40 pigs, including 14 Landrace individuals, and annotated all extracted SNPs using SnpEff version 4.1a (http://snpeff.sourceforge.net/SnpSift.html) [24]. Through this SNP annotation, all SNPs were divided into 31 functional classes, including nsSNPs (Figure 1). Most of the SNPs were located in intergenic or intronic regions; finally, we identified 53,998 nsSNPs (0.205% of the total SNPs). After quality control for all of the nsSNPs, there were 30,822 nsSNPs. Population structure analysis using the genotypic information on these SNPs provided the genetic relationship among breeds. The results from analyzing the population structure clearly distinguished Landrace, Yorkshire, and wild boar (Figure 2).

### nsSNPs in Landrace selective sweep regions

**Figure 4:**
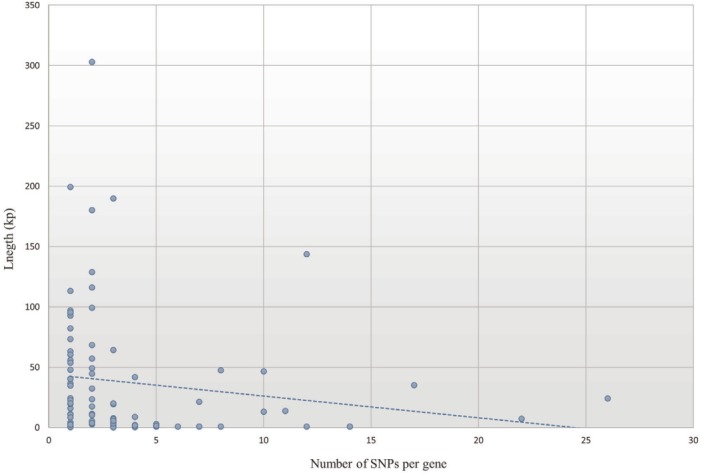
Correlation between length and number of single nucleotide polymorphisms (SNPs) in genes related to non-synonymous SNPs (nsSNPs) in Landrace selective sweep regions.

**Figure 5:**
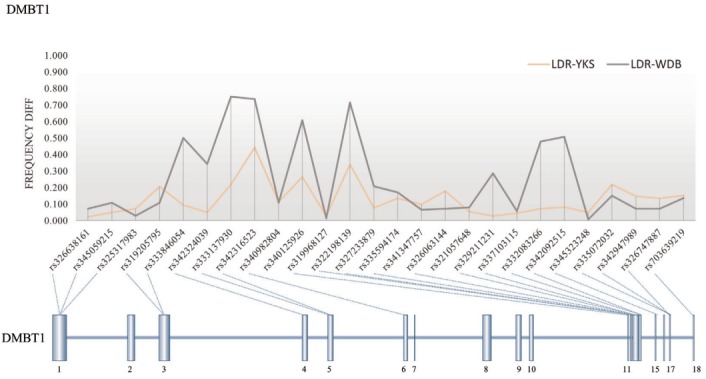
Frequency difference of non-synonymous single nucleotide polymorphisms (nsSNPs) in deleted in malignant brain tumors 1 genes between Landrace and other breeds (Yorkshire and wild boar).

A total of 269 selective sweep regions were obtained from a previous study on the Landrace breed to identify nsSNPs related to selective sweeps [7], and a total of 345 nsSNPs were identified from 55 Landrace selective sweep regions (Figure 3) by re-analyzing the data of previous study resequencing data of Landrace and Yorkshire [7]. Information of 345 nsSNPs in the selective sweep regions of the Landrace genome belonged to 90 genes were shown in Table 1. The average number of nsSNPs per gene was 3.83, and the gene length was not correlated to the number of nsSNPs (Figure 4). The deleted in malignant brain tumors 1 (*DMBT1*) gene consisted of 18 exons harboring 26 nsSNPs that were evenly distributed; this gene had the highest number of nsSNPs among the 90 genes. Moreover, there were considerable frequency differences between Landrace and other breeds (Yorkshire and wild boar) in nsSNPs of the *DMBT1* gene (Figure 5). This suggests that DMBT1 is significantly affected by many nsSNPs in Landrace breed establishment. Previous studies strongly suggested an important role of DMBT1 in the process of fertilization in pigs; it was shown to be secreted in the oviduct and involved in the mechanism of fertilization in porcine species [25,26]. In particular, Ambruosi et al [25] reported that oviduct fluid containing DMBT1 protein was strongly related to the preparation of gametes for fertilization, fertilization itself, and subsequent embryonic development. Therefore, we assumed that nsSNPs of *DMBT1* of Landrace might correlate with the fertilization capacity that was acquired during artificial selection, making the reproductive capacity of Landrace pigs superior to that of other breeds [27].

**Figure 6:**
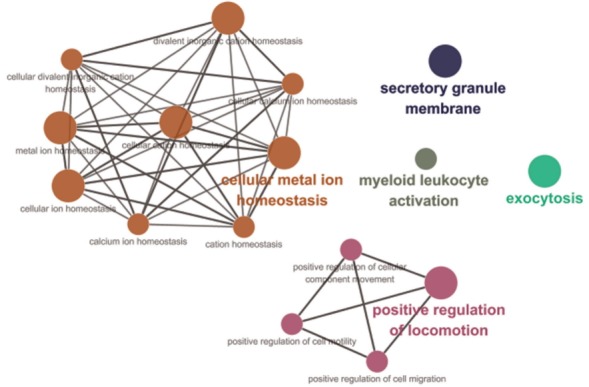
Gene ontology (GO) network analysis of genes related to non-synonymous single nucleotide polymorphisms (SNPs) in Landrace selective sweep regions. Significant results of GO analysis using genes related to non-synonymous SNPs in the selective sweep regions of the Landrace genome with our criteria in ClueGO packages of Cytoscape (number of genes = 4, sharing group percentage = 40.0). These results are largely divided into eight clusters as follows.

Among 90 genes, the functions of 64 genes were predicted, and we performed GO network analysis of these 64 genes using ClueGO [21] to draw inferences on the biological effects of nsSNPs in Landrace selective sweep regions. The information on these networks is shown in Figure 6 and Table 2. The GO network analysis revealed that 19 of the total of 64 genes were associated with five major GO terms, and these major terms were closely related to the reproductive capacity or growth and development of the Landrace breed during the perinatal period. In the GO network, seven genes (C-C motif chemokine ligand 1 [*CCL1*], *CCL23*, hemopexin, mucolipin 1, leucine zipper and EF-hand containing transmembrane protein 2, phospholipase A2 group VI [*PLA2G6*], and protein tyrosine phosphatase, receptor type, C [*PTPRC*]) were related to cellular metal ion homeostasis in seven major GO terms, and this cluster was the largest in this network. Moreover, these terms were similar to the GO results of a positively selected region identified in Wang’s study of Landrace selective sweeps [7]. Metal ions are one major group of mineral; since components of follicular fluid such as Ca, Cu, and Fe significantly increase as the follicles increase in size, some minerals appear to play an important role in pig reproduction [28]. Five genes (ATPase phospholipid transporting 8A1 [*ATP8A1*], *CCL1*, kinesin family member 20B, plasminogen, and *PTPRC*) were shown to be involved in the positive regulation of locomotion, and its network consisted of four GO terms (positive regulation of locomotion, positive regulation of cellular component movement, positive regulation of cell motility, and positive regulation of cell migration). This cellular movement is a central process in the development and maintenance of multicellular organisms. In addition, tissue formation during embryonic development requires the orchestrated movement of cells in a particular direction. It is reasonable to assume that several genes of these four significant GO terms in the selective sweep regions of the Landrace genome might be related to the superior growth and development of Landrace during the perinatal period. Ten genes (*ATP8A1*, bridging integrator 2, CD93 molecule [*CD93*], exophilin 5, GRB2 associated binding protein 2, n-ethylmaleimide-sensitive factor attachment protein, beta, *PLA2G6*, *PTPRC*, and vesicle associated membrane protein 1 [*VAMP1*]) were associated with exocytosis, and five genes (*ATP8A1*, *CD93*, *DMBT1*, *PTPRC*, and *VAMP1*) were classified under the secretory granule membrane term in the GO network. The acrosome contains a single secretory granule and is located in the head of mammalian sperm; secretion from this granule is an absolute requirement for fertilization [29]. Acrosome exocytosis is a synchronized and tightly regulated all-or-nothing process, which provides a unique model for studying the multiple steps of the membrane fusion cascade [29]. Therefore, we assumed that these genes containing nsSNPs in the selective sweep region, which are related to exocytosis and the secretory granule membrane, might have been influenced by artificial selection, considering the distinctive reproductive capacity of the Landrace breed [27].

### Predicting strong effects of nsSNPs on amino acid substitutions in Landrace selective sweep region

Two *in silico* SNP prediction algorithms, SIFT [22] and PolyPhen-2 [23], were applied to estimate the possible effects of the stabilizing residues on protein functions for 345 nsSNPs in Landrace selective sweep regions. The results of SIFT and Polyphen-2 for 345 non-synonymous SNPs are shown in Tables 3, 4.

According to the SIFT analysis, 75 of 345 nsSNPs were classified as being deleterious (for some SNPs, there was low confidence in the findings regarding deleteriousness). PolyPhen-2 calculates the true-positive rate as a fraction of predicted mutations; its results showed that 82 amino acid variants involving nsSNPs in the selective sweep regions of the Landrace genome were likely to exert deleterious functional effects. In addition, 46 of these nsSNPs overlapped with the SIFT results. From the results of the two bioinformatics tools, we reasoned that 46 of the 345 nsSNPs might have strong effects on biological mechanisms during the process of Landrace domestication (Table 4). Forty-six nsSNPs that had strong effects on protein function were distributed among 26 genes and 19 selective sweep regions. In addition, 2:62355986–62756249 among the 55 selective sweep regions containing nsSNPs had the most nsSNPs (37 SNPs), and the results of the two tools for predicting the nsSNP effects showed that 10 of 37 SNPs in 2:62355986–62756249 had strong effects on protein function. This was the largest number of nsSNPs with a strong effect among the total of 55 selective sweep regions of the Landrace genome containing an nsSNP. In addition, three genes belonged to this selective sweep region: ENSSSCG00000013821, ENSSSCG00000013822, and ENSSSCG00000013819. Because the selective region (2: 62355986–62756249) where this gene is located has not been annotated, we estimated the approximate functions of these three genes by analyzing their orthologs. We searched for orthologous genes of these three genes for which the detailed function had been discovered in placental mammals; there were no one-to-one orthologous genes and only many-to-many orthologous genes (Table 5). Because the lists of orthologs of the three genes were the same, we guessed that the functions of the three genes would be very similar. Because the orthologous genes consisted of 18 genes from 8 species from placental mammals and all 18 genes were related to olfactory receptors, we assumed that ENSSSCG00000013821, ENSSSCG 00000013822, and ENSSSCG00000013819 were inferred as olfactory receptors. In a previous study of pig evolution, one of the several significant features of porcine genome expansion involved the olfactory receptor gene family [30]. Martien et al [26] reported that there are 1,301 porcine olfactory receptor genes and 343 partial olfactory receptor genes. This large number of functional olfactory receptor genes most probably reflects the strong reliance of pigs on their sense of smell while scavenging for food. The presence of greater number of nsSNPs in genes related to olfactory receptors suggested important roles of these genes during selection. Additionally, the monoacylglycerol O-acyltransferase 2 (*MOGAT2*) gene was shown to have the greatest number of nsSNPs with a strong effect among the 90 genes. Five SNPs of the total of 11 nsSNPs in the *MOGAT2* gene had strong effects on protein function in this study. Although our GO network analysis did not reveal any particularly important network of *MOGAT2*, this gene has been reported to be important in porcine backfat adipose tissue, which is related to the concentration of lipid and lipid synthesis, as revealed by a transcriptome analysis comparing Landrace and other breeds [31]. In addition, 3 of 26 nsSNPs in the *DMBT1* gene were considered to have strong effects on protein function, as revealed by the SIFT and Polyphen-2 results.

## DISCUSSION

Given the interest of the meat production industry in improving the meat quality or piglet number, a genetic investigation focusing on the selective sweep regions of the Landrace genome was previously performed [7]. This study provided vital information for domestic pig breeding. In most selective sweep studies using whole-genome sequencing data, all SNPs, including nsSNPs, were used to detect selective sweep regions. As nsSNPs are mutations that alter the amino acid sequences of encoded proteins, their presence results in a phenotypic change in the organism. Such changes are usually subjected to natural selection. In the case of Landrace, the domestication process had a shorter generation interval than natural selection. Therefore, we believe that nsSNPs had a diverse evolutionary history during the domestication and artificial selection processes, and advanced studies are required to achieve an accurate interpretation of the Landrace genome using nsSNP information after exploring Landrace positive selection based on whole-genome sequence data. In this study, we performed several analyses of nsSNPs of the Landrace genome to obtain a better understanding of the whole genome. We assumed that the information on these nsSNPs might be associated with novel important biological mechanisms related to particular traits of the Landrace breed. For the precise analysis of the characteristics of the Landrace breed from a genomic perspective, we investigated the biological meaning of nsSNPs in the selective sweep regions of the Landrace genome used in a previous study [7]. As a result, there was no correlation between the number of nsSNPs and gene length per 90 genes containing an nsSNP within the selective sweep regions of the Landrace genome (Figure 5), which was contrary to our expectations. Considering that 22 of 90 genes overlapped with multiple selective sweep regions while the others belonged to a single selective sweep region, we assumed that genes containing many nsSNPs in the selective sweep regions of the Landrace genome were more meaningful than our expectation. Subsequently, based on GO network analysis using genes containing 345 nsSNPs in the selective sweep regions of the Landrace genome, a large proportion of selective sweep regions of the Landrace genome where strong amino acid sequence changes had occurred, were involved in the superior reproductive capacity or growth and development of the Landrace breed during the perinatal period. Some of the GO network results overlapped with the GO analysis of all the selective sweep regions in a previous study, while others involved novel interpretations of the Landrace genome [7].

## CONCLUSION

Our results strongly suggested that Landrace genetic variants, which could give rise to changes in amino acid sequences, might be important factors for the superior reproductive capacity of this breed. We aimed to perform analyses of the Landrace genome using nsSNPs in selective sweep regions. Our results showed that most of the genes affected by nsSNPs in the selective sweep regions may be closely related to the superior reproductive capacity or growth and development of the Landrace breed during the perinatal period. Furthermore, there were indications that nsSNPs in selection had impacted in Landrace breed establishment. This study will provide insights into the impact of the process of domestication on the Landrace genome.

## Figures and Tables

**Table 1:** Gene list containing non-synonymous SNPs in Landrace selective sweep regions

Gene name	CHR	Gene sart	Gene end	# ns SNP	Selective sweep region	Gene name	CHR	Gene sart	Gene end	# ns SNP	Selective sweep region
	
*PLG*	1	8,739,981	8,787,582	8	1:8670943–8797806	*ENSSSCG00000015184*	9	56,925,449	56,927,199	4	9:56869539–57122277
*MELK*	1	265,175,024	265,288,283	1	1:265063188–265212930	*ENSSSCG00000026119*	9	56,962,203	56,963,135	7	
*ZFPL1*	2	6,231,271	6,235,566	1	2:6227731–6239068	*ENSSSCG00000015182*	9	56,971,208	56,972,140	5	
*ENSSSCG00000021162*	2	15,576,680	15,577,609	3	2:15569156–15593980	*ENSSSCG00000028463*	9	56,980,334	56,981,572	5	
*FAM180B*	2	16,204,579	16,206,256	3	2:16111708–16299440	*ENSSSCG00000024117*	9	57,283,042	57,284,501	5	9:57230656–57379772
*ENSSSCG00000025219*	2	62,507,452	62,508,408	1	2:62355986–62756249	*ENSSSCG00000024455*	9	57,293,941	57,296,806	1	
*ENSSSCG00000013821*	2	62,624,616	62,625,548	14		*DMTF1*	9	102,893,256	102,929,921	1	9:102847568–103896296
*ENSSSCG00000013822*	2	62,644,870	62,645,796	14		*DENND1B*	10	25,096,498	25,193,569	1	10:25139986–25249094
*ENSSSCG00000013819*	2	62,669,703	62,670,662	8		*ENSSSCG00000010907*	10	26,249,079	26,284,300	17	10:26197521–26710943
*MCOLN1*	2	72,056,664	72,151,713	1	2:72143419–72172550	*PTPRC*	10	26,308,759	26,332,284	2	10:26197521–26710943
*ENSSSCG00000014078*	2	85,731,838	85,732,242	4	2:85467258–86506548	*KIAA1462*	10	45,386,450	45,428,443	4	10:45403837–45436342
*ANKRD31*	2	85,774,886	85,807,199	2		*GJD4*	10	63,677,681	63,683,060	2	10:63669866–63725092
*ANKDD1B*	2	86,257,325	86,321,705	3		*ENSSSCG00000021829*	11	11,141,413	11,236,840	1	11:10400737–11376721
*SDK1*	3	3,634,288	3,824,252	3	3:3730382–3773007	*ENSSSCG00000020699*	11	11,355,261	11,378,042	1	
*PLA2G6*	5	6,996,414	7,059,756	1	5:6988526–7058468	*CCDC168*	11	78,361,372	78,368,847	22	11:78318648–78678168
*BIN2*	5	17,315,117	17,339,457	1	5:17248525–17487183	*DNAI2*	12	6,779,152	6,799,278	3	12:6771152–6805468
*TAC3*	5	24,048,553	24,056,427	3	5:23288996–24074802	*MARCH10*	12	15,897,681	15,944,341	10	12:15890650–15938045
*ZBTB39*	5	24,066,660	24,068,784	4		*MAPT*	12	17,123,471	17,172,747	2	12:16937097–17191735
*NCAPD2*	5	66,432,584	66,443,844	1	5:66396846–66725591	*CCL23*	12	41,160,877	41,165,234	3	12:41158920–41165901
*VAMP1*	5	66,646,135	66,647,743	1		*CCL1*	12	42,467,618	42,471,014	3	12:42468535–42621081
*TAPBPL*	5	66,647,211	66,658,624	2		*ENSSSCG00000017834*	12	50,542,085	50,552,985	1	12:50535159–50581774
*DMBT1*	6	43,728,925	43,753,137	26	6:43719388–43757067	*SHPK*	12	51,572,871	51,592,551	1	12:51579885–51586595
*ENSSSCG00000027618*	6	119,199,612	119,199,920	3	6:119198939–119344591	*SPNS3*	12	52,389,071	52,445,090	1	12:52401285–52444137
*MCOLN2*	6	119,212,826	119,273,364	1		*CCDC66*	13	42,284,163	42,341,496	2	13:41196871–42465605
*PCNX1*	7	100,745,867	100,862,081	2	7:100703442–100775415	*NOC4L*	14	24,724,492	24,730,021	2	14:24592939–24779049
*PLD4*	7	131,340,863	131,347,987	3	7:131291714–131388688	*DDX51*	14	24,730,045	24,732,878	2	
*ENSSSCG00000002551*	7	131,356,311	131,359,461	5		*EP400*	14	24,748,336	24,847,567	2	
*ATP8A1*	8	35,180,992	35,309,867	2	8:34998191–35275833	*ENSSSCG00000010013*	14	50,652,381	50,652,947	1	14:50647172–50719083
*ENSSSCG00000027999*	9	2,277,256	2,278,264	7	9:2223331–2577505	*OSBP2*	14	50,669,019	50,849,290	2	
*OVCH2*	9	2,307,953	2,321,197	10		*KIF20B*	14	110,499,118	110,581,337	1	14:110280822–110542445
*ENSSSCG00000025898*	9	2,361,209	2,362,147	5		*FGFR1IIIC*	15	55,215,592	55,269,381	1	15:55142754–55608192
*ENSSSCG00000023477*	9	2,370,889	2,371,830	12		*LETM2*	15	55,274,276	55,294,333	1	
*ENSSSCG00000029634*	9	2,455,370	2,528,783	1		*WHSC1L1*	15	55,338,007	55,406,429	2	
*TRIM3*	9	3,923,986	3,940,046	1	9:3927497–3978728	*DDHD2*	15	55,414,565	55,455,195	1	
*HPX*	9	3,946,381	3,955,253	4		*ASH2L*	15	55,512,104	55,552,504	1	
*SMPD1*	9	3,961,589	3,964,504	1		*ENSSSCG00000029683*	15	128,593,493	128,594,377	6	15:128498493–128627886
*MOGAT2*	9	11,119,062	11,132,962	11	9:11120076–11136889	*CWC27*	16	46,572,512	46,875,541	2	16:46472193–46771773
*THAP12*	9	11,652,415	11,669,844	2	9:11449284–11760977	*CD93*	17	34,381,626	34,384,902	2	17:34206246–34400408
*GAB2*	9	13,936,307	14,135,685	1	9:13934282–14030509	*GZF1*	17	34,441,517	34,447,221	3	17:34421087–34505222
*ELMOD1*	9	40,189,956	40,282,814	1	9:40189621–40286365	*NAPB*	17	34,450,368	34,485,152	1	
*ATM*	9	40,925,895	40,945,439	3	9:40793693–41170478	*CSTL1*	17	34,492,910	34,496,585	2	
*KDELC2*	9	41,043,564	41,065,077	7		*CST7*	17	34,906,655	34,915,135	1	17:34901568–34908632
*EXPH5*	9	41,073,546	41,217,329	12		*DEFB119*	17	39,921,302	39,931,655	2	17:39862221–40018288
*ENSSSCG00000023913*	9	41,145,017	41,152,176	3		*DEFB116*	17	39,996,662	39,999,076	1	
*ARHGAP20*	9	43,174,648	43,222,583	1	9:43134418–43291918	*ENSSSCG00000007337*	17	46,357,154	46,401,936	2	17:46275105:46424519

SNPs, single nucleotide polymorphisms; nsSNPs, non-synonymous SNPs.

We show the information of genes containing non-synonymous SNPs. In this table, the fifth column indicates the number of non-synonymous SNPs in each gene and the seventh column presents information on the selective sweep regions of the Landrace genome and selective sweep name, consisting of chromosome, start position, and end position.

**Table 2:** Information of gene ontology (GO) network analysis of genes related to non-synonymous SNPs in Landrace selective sweep regions

GO ID	GO Term	Term p-value	Group p-value	#Genes	Associated genes found
GO:0002274	Myeloid leukocyte activation	0.005	0.005	7	ATP8A1, BIN2, CD93, GAB2, MAPT, PTPRC, SHPK
GO:0006887	Exocytosis	0.001	0.001	10	ATP8A1, BIN2, CD93, EXPH5, GAB2, NAPB, PLA2G6, PLG, PTPRC, VAMP1
GO:0030667	Secretory granule membrane	0.003	0.003	5	ATP8A1, CD93, DMBT1, PTPRC, VAMP1
GO:0040017	Positive regulation of locomotion	0.016	0.017	5	ATP8A1, CCL1, KIF20B, PLG, PTPRC
GO:0051272	Positive regulation of cellular component movement	0.013		5	ATP8A1, CCL1, KIF20B, PLG, PTPRC
GO:2000147	Positive regulation of cell motility	0.012		5	ATP8A1, CCL1, KIF20B, PLG, PTPRC
GO:0030335	Positive regulation of cell migration	0.010		5	ATP8A1, CCL1, KIF20B, PLG, PTPRC
GO:0006873	Cellular ion homeostasis	0.003	0.006	7	CCL1, CCL23, HPX, LETM2, MCOLN1, PLA2G6, PTPRC
GO:0055080	Cation homeostasis	0.005		7	CCL1, CCL23, HPX, LETM2, MCOLN1, PLA2G6, PTPRC
GO:0030003	Cellular cation homeostasis	0.003		7	CCL1, CCL23, HPX, LETM2, MCOLN1, PLA2G6, PTPRC
GO:0055065	Metal ion homeostasis	0.003		7	CCL1, CCL23, HPX, LETM2, MCOLN1, PLA2G6, PTPRC
GO:0072507	Divalent inorganic cation homeostasis	0.015		5	CCL1, CCL23, MCOLN1, PLA2G6, PTPRC
GO:0006875	Cellular metal ion homeostasis	0.001		7	CCL1, CCL23, HPX, LETM2, MCOLN1, PLA2G6, PTPRC
GO:0072503	Cellular divalent inorganic cation homeostasis	0.013		5	CCL1, CCL23, MCOLN1, PLA2G6, PTPRC
GO:0055074	Calcium ion homeostasis	0.011		5	CCL1, CCL23, MCOLN1, PLA2G6, PTPRC
GO:0006874	Cellular calcium ion homeostasis	0.010		5	CCL1, CCL23, MCOLN1, PLA2G6, PTPRC

SNPs, single nucleotide polymorphisms.

Significant results of GO analysis using genes related to non-synonymous SNPs in the selective sweep regions of the Landrace genome with our criteria in ClueGO packages of Cytoscape (number of genes = 4, sharing group percentage = 40.0). These results are largely divided into eight clusters as follows.

**Table 3:** Summary of non-synonymous single amino acid variation in genes of Landrace selective sweep using SIFT and Polyphen-2

		Polyphen-2			
		
		Benign	Possibly damaging	Probably damaging	Total
SIFT	Deleterious	29	19	27	75
	Tolerated	234	21	15	270
	Total	263	40	42	345

SIFT, sorting intolerant from tolerant; Polyphen-2, polymorphism phenotyping v2.

**Table 4:** Forty-six non-synonymous SNPs with strong effects on protein functions based on SIFT and Polyphen-2

SNP	CHR	POS	A1	A2	SIFT prediction	SIFT score	Polyphen-2 prediction	Polyphen-2 score	Gene	Selective sweep
rs328613228	2	16,206,079	T	G	deleterious	0	probably damaging	0.997	*FAM180B*	2:16111708:16299440
2:62624837	2	62,624,837	G	A	deleterious	0.017	possibly damaging	0.853	*ENSSSCG00000013821*	2:62355986:62756249
rs340857214	2	62,625,107	G	A	deleterious	0.021	possibly damaging	0.539		
2:62625190	2	62,625,190	A	T	deleterious	0.028	possibly damaging	0.934		
rs335820735	2	62,644,986	A	T	deleterious	0.008	probably damaging	0.999	*ENSSSCG00000013822*	
rs343007761	2	62,645,014	T	G	deleterious	0.018	possibly damaging	0.506		
2:62645060	2	62,645,060	A	G	deleterious	0.012	possibly damaging	0.604		
rs325197977	2	62,645,081	A	G	deleterious	0	possibly damaging	0.934		
2:62669920	2	62,669,920	G	A	deleterious	0.008	possibly damaging	0.934	*ENSSSCG00000013819*	
2:62669953	2	62,669,953	T	G	deleterious	0.007	possibly damaging	0.934		
2:62670031	2	62,670,031	G	A	deleterious	0.012	probably damaging	0.999		
rs342394815	2	85,732,226	T	C	deleterious	0.002	probably damaging	0.999	*ENSSSCG00000014078*	2:85467258:86506548
rs337260402	2	85,732,237	T	G	deleterious	0.003	probably damaging	0.97		
rs326720643	2	85,775,718	A	G	deleterious	0.007	probably damaging	0.984	*ANKRD31*	
rs318473425	2	86,321,677	T	A	deleterious	0.033	probably damaging	0.995	*ANKDD1B*	
rs329106718	5	66,654,214	C	T	deleterious	0	probably damaging	0.993	*TAPBPL*	5:66396846:66725591
rs326638161	6	43,729,346	T	C	deleterious	0.007	probably damaging	0.988	*DMBT1*	6:43719388:43757067
rs322198139	6	43,750,820	G	T	deleterious	0.017	possibly damaging	0.915		
rs321057648	6	43,750,963	A	G	deleterious	0.009	possibly damaging	0.663		
6:119199835	6	119,199,835	T	A	deleterious	0.006	probably damaging	0.998	*ENSSSCG00000027618*	6:119198939:119344591
rs327779736	8	35,181,016	A	T	deleterious	0	possibly damaging	0.944	*ATP8A1*	8:34998191:35275833
rs81399633	8	35,181,037	A	G	deleterious	0.023	possibly damaging	0.896		
rs343636299	9	2,311,094	T	C	deleterious	0.042	probably damaging	1	*OVCH2*	9:2223331:2577505
rs318298009	9	3,930,944	T	A	deleterious	0.006	probably damaging	0.996	*TRIM3*	9:3927497:3978728
9:11129485	9	11,129,485	T	G	deleterious	0.035	probably damaging	0.995	*MOGAT2*	9:11120076:11136889
rs340556206	9	11,129,936	T	C	deleterious	0.013	probably damaging	0.999		
rs81509118	9	11,130,742	A	G	deleterious	0.036	probably damaging	1		
rs342457070	9	11,130,778	C	A	deleterious	0.005	probably damaging	0.991		
rs327337551	9	11,130,783	G	C	deleterious	0.047	possibly damaging	0.697		
rs338381437	9	11,666,878	G	A	deleterious	0.003	probably damaging	0.983	*THAP12*	9:11449284:11760977
rs81214615	9	41,047,573	T	A	deleterious	0.024	probably damaging	0.99	*KDELC2*	9:40793693:41170478
rs339385194	9	41,076,701	G	T	deleterious	0.04	probably damaging	0.999	*EXPH5*	
9:56962342	9	56,962,342	A	G	deleterious	0.028	possibly damaging	0.616	*ENSSSCG00000026119*	9:56869539:57122277
9:56962578	9	56,962,578	A	C	deleterious	0.026	probably damaging	0.994		
rs328160175	9	56,971,732	G	A	deleterious	0.016	probably damaging	0.994	*ENSSSCG00000015182*	
rs335643554	9	56,980,378	C	T	deleterious	0.032	possibly damaging	0.539	*ENSSSCG00000028463*	
rs331490061	9	56,981,034	A	G	deleterious	0.004	possibly damaging	0.927		
rs326014276	10	63,681,709	G	C	deleterious	0.037	possibly damaging	0.944	*GJD4*	10:63669866:63725092
rs339353031	11	78,365,823	G	A	deleterious	0.008	probably damaging	0.983	*CCDC168*	11:78318648:78678168
11:78367889	11	78,367,889	G	A	deleterious	0	probably damaging	0.993		
rs342686832	11	78,367,955	A	G	deleterious	0.034	possibly damaging	0.94		
rs325650226	12	15,917,860	T	C	deleterious	0.002	probably damaging	0.999	*MARCH10*	12:15890650:15938045
rs336224471	12	15,917,910	A	C	deleterious	0.03	possibly damaging	0.82		
15:55400479	15	55,400,479	A	G	deleterious	0.032	probably damaging	1	*WHSC1L1*	15:55142754:55608192
rs339461760	16	46,612,542	C	G	deleterious	0.007	probably damaging	0.998	*CWC27*	16:46472193:46771773
rs324424231	17	46,357,195	A	G	deleterious	0	probably damaging	0.998	*ENSSSCG00000007337*	17:46275105:46424519

SNPs, single nucleotide polymorphisms; SIFT, sorting intolerant from tolerant; Polyphen-2, polymorphism phenotyping v2.

We identified that 46 of 345 non-synonymous SNPs in the selective sweep regions of the Landrace genome had strong effects on protein function as determined with both in silico tools: SIFT and PolyPhen-2.

**Table 5:** Information on the orthologs of three genes (ENSSSCG00000013821, ENSSSCG00000013822, and ENSSSCG000000138149) in selective sweep 2:62355986–62756249

Species	Match gene symbol	Match ensemble gene ID	Compare regions	ENSSSCG00000013821			ENSSSCG00000013822			ENSSSCG00000013819		
		
				dN/dS	Target %id	Query %id	dN/dS	Target %id	Query %id	dN/dS	Target %id	Query %id
Chimpanzee (Pan troglodytes)	OR7A5	*ENSPTRG00000010603*	19:15,130,772–15,137,945	0.350	69.0	70.7	0.372	69.6	71.8	0.327	71.2	70.9
Chimpanzee (Pan troglodytes)	OR7A10	*ENSPTRG00000010604*	19:15,143,753–15,144,682	0.377	70.6	70.1	0.333	71.8	71.8	0.338	71.8	69.4
Gibbon (Nomascus leucogenys)	OR7A17	*ENSNLEG00000005159*	GL397382.1:231,228–275,098	0.383	71.0	70.7	0.359	70.3	70.6	0.290	73.2	70.9
Gorilla (Gorilla gorilla gorilla)	OR7A10	*ENSGGOG00000015049*	19:15,120,105–15,121,034	-	70.6	70.1	-	70.9	70.9	-	72.2	69.7
Gorilla (Gorilla gorilla gorilla)	OR7A17	*ENSGGOG00000034834*	19:15,160,189–15,161,115	-	72.5	72.0	-	72.8	72.8	-	73.1	70.6
Human (Homo sapiens)	OR7A10	*ENSG00000127515*	19:14,840,948–14,841,877	0.418	70.2	69.8	0.377	70.6	70.6	0.361	71.8	69.4
Human (Homo sapiens)	OR7A17	*ENSG00000185385*	19:14,880,426–14,881,452	0.338	72.2	71.7	0.356	72.5	72.5	0.317	72.5	70.0
Human (Homo sapiens)	OR7A5	*ENSG00000188269*	19:14,792,490–14,835,376	0.354	69.6	71.4	0.370	70.2	72.5	0.313	71.5	71.3
Mouse (Mus musculus)	Olfr1353	*ENSMUSG00000042774*	10:78,963,309–78,971,338	-	62.5	62.1	-	61.2	61.2	0.243	65.1	62.8
Mouse (Mus musculus)	Olfr1352	*ENSMUSG00000046493*	10:78,981,050–78,987,903	0.238	68.6	68.2	0.224	67.3	67.3	-	68.6	66.3
Mouse (Mus musculus)	Olfr19	*ENSMUSG00000048101*	16:16,672,228–16,676,405	0.245	68.3	67.9	0.267	66.3	66.3	0.253	67.6	65.3
Mouse (Mus musculus)	Olfr57	*ENSMUSG00000060205*	10:79,028,741–79,036,274	0.308	66.5	68.2	0.289	64.3	66.3	0.349	65.2	65.0
Mouse (Mus musculus)	Olfr1351	*ENSMUSG00000063216*	10:79,012,472–79,019,645	0.308	64.6	66.2	0.303	62.1	64.1	0.345	64.3	64.1
Mouse (Mus musculus)	Olfr8	*ENSMUSG00000094080*	10:78,950,636–78,958,378	0.284	63.2	63.0	0.317	58.4	58.6	-	60.7	58.8
Mouse (Mus musculus)	Olfr1354	*ENSMUSG00000094673*	10:78,913,171–78,920,399	0.264	63.6	63.3	-	59.0	59.2	-	62.3	60.3
Orangutan (Pongo abelii)	OR7A5	*ENSPPYG00000009655*	19:15,004,902–15,005,858	0.373	67.9	69.5	0.395	67.3	69.3	0.351	68.9	68.4
Orangutan (Pongo abelii)	OR7A10	*ENSPPYG00000009656*	19:15,019,264–15,020,193	0.453	69.6	69.1	0.402	70.9	70.9	0.350	71.2	68.8
Orangutan (Pongo abelii)	OR7A17	*ENSPPYG00000009658*	19:15,062,903–15,091,843	0.344	70.9	70.4	0.339	71.5	71.5	0.342	70.9	68.4
Rat (Rattus norvegicus)	Olr1073	*ENSRNOG00000031688*	7:13,378,338–13,379,273	-	62.1	62.1	-	61.7	62.1	0.270	65.3	63.4
Rat (Rattus norvegicus)	Olr1076	*ENSRNOG00000039448*	7:13,424,355–13,425,311	0.263	66.0	67.5	0.248	63.8	65.7	0.285	64.8	64.4
Rat (Rattus norvegicus)	Olr1075	*ENSRNOG00000039449*	7:13,403,899–13,404,858	0.290	67.1	68.8	0.272	66.1	68.3	0.291	67.4	67.2
Rat (Rattus norvegicus)	Olr1085	*ENSRNOG00000047090*	7:13,673,934–13,674,866	-	63.2	63.0	0.343	58.4	58.6	0.327	62.3	60.3
Rat (Rattus norvegicus)	Olr1079	*ENSRNOG00000049781*	7:13,488,205–13,489,137	0.276	63.6	63.3	0.395	59.4	59.6	0.336	62.6	60.6
Rat (Rattus norvegicus)	Olr1077	*ENSRNOG00000054107*	7:13,460,476–13,461,405	0.229	69.3	68.8	0.241	67.0	67.0	0.236	68.0	65.6
Rat (Rattus norvegicus)	Olr1082	*ENSRNOG00000058943*	7:13,553,010–13,553,963	0.279	61.8	63.0	0.348	58.0	59.6	0.342	59.6	59.1
Rat (Rattus norvegicus)	Olr1083	*ENSRNOG00000061480*	7:13,587,479–13,588,411	0.290	63.2	63.0	0.352	60.3	60.5	0.332	62.6	60.6
Vervet-AGM (Chlorocebus sabaeus)	OR7A10	*ENSCSAG00000006193*	6:13,469,888–13,471,167	0.347	70.2	69.8	0.330	72.2	72.2	0.348	71.8	69.4
